# Use of the Term “Atypical Hepatic Hemangioma” in Radiology Reports: An Analysis of Diagnostic Precision

**DOI:** 10.3390/jcm15155923

**Published:** 2026-07-29

**Authors:** Shlomit Tamir, Tomer Krutik, Ran Kedem Mashraki, Ilan Shelef, Eli Atar, Ahuva Grubstein, Jacob Sosna, Gal Ben-Arie

**Affiliations:** 1 Department of Radiology, Rabin Medical Center, Gray Faculty of Medicine and Health Sciences, Tel Aviv University, Zeev Jabotinsky St. 39, Petah Tikva 4941492, Israel; 2 Faculty of Health Sciences, Ben Gurion University of the Negev, Yitzhack I. Rager Blvd 151, Be’er Sheva 84101, Israel; 3 Department of Radiology, Soroka Medical Center, Faculty of Health Sciences, Ben Gurion University of the Negev, Yitzhack I. Rager Blvd 151, Be’er Sheva 84101, Israel; 4 Department of Radiology, Hadassah Medical Center, Faculty of Medicine, Hebrew University of Jerusalem, Kalman Ya’akov Man St., Jerusalem 9112001, Israel

**Keywords:** hemangioma, atypical, hepatic lesions, missed malignancy, unnecessary workup

## Abstract

**Background/Objectives**: Hepatic hemangiomas are the most common benign liver tumors, typically diagnosed confidently by their classic imaging features. However, a subset displays atypical characteristics that complicate diagnosis. We hypothesized that the term “atypical hemangioma” is used inconsistently and may contribute to both over-investigation of benign lesions and missed malignancies. Accordingly, this study aimed to assess the implications of the use of this term in radiology reports. **Methods**: This retrospective multicenter study reviewed 327 hepatic lesions labeled as atypical hemangiomas in radiology reports from two tertiary hospitals between 2013 and 2023. Two abdominal radiologists independently assessed imaging characteristics. Final diagnoses were based on imaging stability, histopathology or clinical follow-up. **Results**: Of the 327 lesions, 305 (93.3%) were benign and 22 (6.7%) malignant. Among the malignant lesions, 11 (50%) had no recommendations for further workup, and 10 (45%) did not mention malignancy in the differential diagnosis. Malignant lesions were significantly larger (mean size: 49.5–50.8 mm vs. 28.1–29.1 mm; *p* < 0.001), more likely to have ill-defined margins (50–54.5% vs. 17.9–18.1%; *p* ≤ 0.001), early heterogeneous enhancement (46.2% vs. ~9.5%; *p* < 0.001), and restricted diffusion (75–100% vs. 7.8–17.6%; *p* < 0.001). Among the benign lesions, 128 (42%) could have been confidently diagnosed at baseline, yet 62 of these lesions underwent unnecessary additional workup. **Conclusions**: The term “atypical hemangioma” is inconsistently used and associated with both delayed cancer diagnosis and excessive workup of benign lesions. Standardized terminology and risk-adapted strategies may improve diagnostic accuracy, optimize management, and minimize patient harm. Prospective validation of standardized imaging criteria is warranted.

## 1. Introduction

Hepatic hemangiomas are the most common benign liver tumors, with reported incidence ranging from 1% to 20% in the general population. These lesions, composed of clusters of blood-filled cavities lined by endothelial cells, are typically classified as either capillary or cavernous based on their microscopic structure. Liver hemangiomas are often asymptomatic, discovered incidentally, and can range from a few millimeters to over 10 cm in size [1,2,3,4].

The classic imaging appearance of hepatic hemangiomas includes homogeneously hyperechoic masses with well-defined margins and minimal or no internal blood flow on Doppler ultrasound (US) [1,2,3]. On unenhanced computed tomography (CT), hemangiomas typically appear hypoattenuating, while magnetic resonance imaging (MRI) classically demonstrates markedly high signal intensity on T2-weighted images [2,4,5]. On contrast-enhanced CT and MRI, hemangiomas exhibit a characteristic peripheral nodular discontinuous enhancement, progressing centripetally over time, a pattern highly specific for diagnosis [2,3]. These classic imaging characteristics enable confident diagnosis, making biopsy and follow-up unnecessary [2,3,4].

Nevertheless, a subset of hepatic hemangiomas displays imaging features that differ from the typical pattern and are commonly termed “Atypical Hemangiomas”. These non-classical features include heterogeneous echotexture, ill-defined borders, rapid or incomplete enhancement, and occasional calcifications [2,3]. Larger hemangiomas, in particular, are more likely to demonstrate non-classical characteristics due to internal changes such as necrosis, hemorrhage, or fibrosis [2,4]. The presence of non-classical features may prompt radiologists to recommend additional imaging, follow-up studies, or even biopsies, raising concerns about unnecessary interventions [1,3].

In other cases, malignant lesions not fully characterized may be termed “atypical hemangiomas”, thus leading to delayed cancer diagnosis [6,7]. Indeed, the term “atypical hemangioma” is used inconsistently across the literature, often as a catch-all descriptor for lesions that do not conform to the classic imaging criteria [1,3]. This vagueness can lead to inconsistency among radiologists, overuse of the term and subsequent misdiagnosis of malignant or benign lesions [1,3,7]. Given that abdominal imaging studies are frequently interpreted by radiologists without specific hepatobiliary expertise, precise and standardized terminology is particularly important to reduce interpretive variability and support appropriate patient management [8,9,10,11,12].

In this study, we investigated the characteristics and final diagnoses of lesions labeled as “atypical hemangiomas” in radiology reports and evaluated whether the use of this reporting term was associated with unnecessary imaging studies and misclassification of malignant lesions as benign. For this study, we distinguish between the reporting label and the final diagnostic entity. We use the phrase “lesions labeled as atypical hemangiomas” to describe all lesions assigned this term in the original radiology report, regardless of their eventual diagnosis. We reserve the term “atypical hemangioma” for lesions ultimately confirmed as hemangiomas that lacked the classic imaging characteristics of a hepatic hemangioma on the index examination.

## 2. Methods

This retrospective study was approved by the Institutional Review Board of the two largest tertiary referral centers in the country’s largest Health Maintenance Organization (HMO). We searched all abdominal radiology US, CT and MRI examinations performed at both institutions between 2013 and 2023 for the term “Atypical Hemangioma,” using a predefined lexicon that included multiple spelling variations and synonymous phrases. Examinations in which the reporting radiologist used this term for a lesion, either as the sole diagnosis or as part of the differential diagnosis, were designated as “index studies.” Patients were eligible if they were 18 years or older.

**Figure 1:**
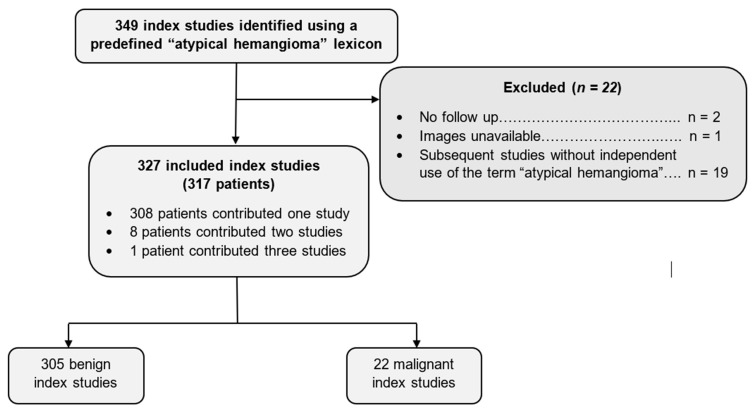
Flowchart of study selection. The unit of inclusion was the imaging study. When the same lesion was labelled as “atypical hemangioma” on more than one examination, each examination was considered an independent index study. Subsequent examinations in which the term appeared only in the referral/history but was not used by the reporting radiologist in the differentia, were excluded.

The unit of inclusion was the imaging study, not the patient or the lesion. Each study was evaluated independently based on the terminology used in its own radiology report. Accordingly, if the same lesion was labeled “atypical hemangioma” (or a synonymous term) on more than one examination—for example, ultrasound followed by CT—each examination was included as a separate index study. Conversely, subsequent examinations in which “atypical hemangioma” appeared only in the referral or clinical history, but was not used by the reporting radiologist in that examination’s own differential diagnosis, were not considered eligible index studies. Inclusion was therefore based solely on the terminology used in the original radiology report and did not imply that the lesion was ultimately a hemangioma. Accordingly, we refer to the full study cohort as “lesions labeled as atypical hemangiomas.” Patients were excluded if the available imaging, pathological, and clinical data were insufficient to establish a final lesion classification or if the relevant imaging studies were unavailable (Figure 1). Final lesion classification was established independently of the original reporting label, using the composite reference standard described below.

Study data were collected and managed using REDCap electronic data capture tools. REDCap (Research Electronic Data Capture, version 15.0.12) is a secure, web-based software platform designed to support data capture for research studies [13,14].

The report of the index study was reviewed to document any additional diagnoses mentioned in the differential diagnosis of the report, as well as whether any additional steps were recommended for further workup, including additional imaging studies, biopsies, or surgical and medical consultations.

Two fellowship-trained abdominal radiologists with 12 (GBA, Reader 1, R1) and 16 (ST, Reader 2, R2) years of experience independently reviewed the index studies in the shared HMO’s PACS (Carestream Health©), blinded to other imaging studies and to clinical data. For CT examinations, all available phases were reviewed. For MRI examinations, T2 signal intensity was interpreted in conjunction with enhancement characteristics and diffusion findings on DWI and corresponding ADC maps. Imaging characteristics of the lesion and the background liver were documented according to the modality used. Modality-specific features were assessed only when applicable: echogenicity was evaluated on ultrasound, enhancement characteristics and washout were evaluated on contrast-enhanced studies and diffusion restriction was evaluated on MRI. Imaging features were recorded only when assessable for the corresponding imaging modality; therefore, denominators vary across individual imaging characteristics. Prior to separately reviewing the cases, the two radiologists reviewed 15 cases together, 5 from each modality, to establish an agreement on categorization of each feature. Discrepancies between the readers were not adjudicated; instead, each reader’s independent assessment was retained and analyzed separately. Inter-rater agreement between the two readers was assessed for both categorical and continuous imaging variables. For categorical variables, agreement was calculated using weighted kappa statistics, with values interpreted as poor (<0.20), fair (0.21–0.40), moderate (0.41–0.60), good (0.61–0.80), or excellent (>0.80) agreement. For lesion size, a continuous variable, agreement was assessed using the intraclass correlation coefficient (ICC), calculated with a two-way mixed-effects model and an absolute agreement definition, with ICC values interpreted as poor (<0.50), moderate (0.50–0.75), good (0.75–0.90), or excellent (>0.90). Final lesion classification was based on the composite reference standard and was determined by consensus between the two readers.

Lesion features assessed included size and non-classical characteristics such as margins (ill-defined borders), partial or complete hypoechogenicity on ultrasound, and enhancement characteristics on CT/MRI (peripheral continuous, early homogeneous, early heterogeneous enhancement that was not in the form of classic peripheral nodules, absence of the expected progressive centripetal filling of contrast on consecutive phases, and early washout), as well as restricted diffusion (Table 1), which was assessed qualitatively by reviewing diffusion-weighted images together with the corresponding apparent diffusion coefficient (ADC) maps and was recorded only when increased signal on high-b-value diffusion-weighted images corresponded to decreased signal on the ADC map. Background liver characteristics included the presence of additional suspicious lesions, moderate to severe hepatic steatosis, and moderate to severe liver fibrosis (Table S1, Supplementary Data). Following review of the index study, the radiologists reviewed prior available studies to determine whether the lesion could be classified as benign at the time of the index study. Finally, ground truth results were categorized based on additional imaging studies including follow-up examinations with US, CT, PET-CT, MRI and TC 99 m red blood cell scans, as well as pathological reports and clinical data from the electronic medical record (EMR), including diagnosis of any malignant disease. The composite reference standard could include characteristic imaging findings on the index or subsequent examinations, interval imaging stability, histopathological findings, and longitudinal clinical information from the electronic medical record. Lesions that remained indeterminate on the index examination were not classified as probably benign without subsequent imaging or clinical follow-up.

Lesions were classified as benign if they demonstrated a typical classical appearance of hemangiomas as detailed in the introduction, or other known benign entities (e.g., FNH, abscess) on imaging studies or histopathology. Hemangiomas were further classified as giant hemangioma, slow-filling hemangioma, flash-filling hemangioma, or hemangioma with no unusual features. Giant hemangiomas were defined as hemangiomas exceeding 4 cm. Flash-filling hemangiomas were defined as small lesions showing homogeneous arterial-phase enhancement without washout and persistent enhancement similar to the blood pool on subsequent phases. This classification was applied only in patients without a known extrahepatic primary malignancy or cirrhosis or moderate-to-severe hepatic fibrosis, and stability on prior or follow-up imaging was required. In cases where the lesion could not be fully characterized, lesions were classified as probably benign if follow-up imaging demonstrated stability or if medical records indicated that the patient was alive without any malignancy for over two years after the index study.

Malignant lesions were determined by a pathological report when biopsy or excision was performed, or when a malignant diagnosis was confirmed and the lesion demonstrated growth on follow-up studies.

## 3. Results

### 3.1. Study Population

A total of 349 index studies were identified from the Radiological Information System (RIS). Multiple index studies of a single patient were included only when the relevant term from the predefined lexicon was used in the differential diagnosis in each of the included studies. After excluding 22 studies due to lack of follow-up or missing images, 327 index studies of 317 patients (163 females, 164 males; mean age 58 years, range 20–95, SD 16.4) were included in the analysis (Figure 1). Eight patients had two index studies each, and one patient had three index studies. Since each index study included one lesion labeled as an ‘atypical hemangioma’, a total of 327 labeled lesions were evaluated across 327 index studies. Because the unit of inclusion was the index study rather than the underlying lesion, in nine patients (eight with two qualifying studies and one with three) the same underlying lesion was independently labeled ‘atypical hemangioma’ on more than one examination and therefore contributed more than one observation to the analysis. All subsequent analyses (Section 3.4, Section 3.5 and Section 3.6) were therefore performed at the study/lesion level; patient-level counts are reported here solely to characterize the cohort’s demographic composition (Table 2) and to account for the difference between the number of studies and the number of patients. The index studies consisted of 106 US, 165 CT, and 56 MRI scans (Table 2). The studies were originally interpreted by 51 attending radiologists across both institutions, with 196 studies (59.9%) performed at X Medical Center and 131 (40.1%) at Y Medical Center.

### 3.2. Index Study Report

Out of the 327 index studies, 213 (65.1%) were reported as atypical hemangiomas without alternative diagnoses. In 98 studies (30%), other benign diagnoses, such as FNH, adenoma, fat deposition, or cysts, were mentioned in the differential diagnosis. A malignant diagnosis was suggested in addition to atypical hemangioma in 32 studies (9.8%). In 16 studies (4.8%), both benign and malignant entities were suggested.

Additional diagnostic workup was recommended in 172 studies (52.6%), with two strategies suggested in 21 cases and three in 4 cases. Recommendations included: 52 US, 43 CT, 69 MRI, 4 PET-CT, and 13 RBC-TC scans, in addition to 1 laboratory test and 19 specialty consultations. Biopsy was not recommended in any of the index studies.

### 3.3. Ground Truth

Longitudinal clinical information from the electronic medical record was available for 196 studies (59.9%), with a mean follow-up duration of 58 months and a median 50 months. This figure refers specifically to clinical follow-up and not to all components of the composite reference standard. Additional imaging was available in 144 cases (including 15 US examinations, 65 CT scans, 50 MRI scans, 8 PET-CTs, and 6 RBC-TC scans), and histopathological confirmation was available in 17 cases; these categories were not mutually exclusive. For studies without separate longitudinal clinical documentation, final classification was established using characteristic imaging findings on the index examination, additional imaging, and/or histopathology, as applicable. Patient demographics and imaging modalities are summarized in Table 2. Based on a composite review of clinical, imaging, and pathology data, 117 lesions (35.8%) were classified as hemangiomas, 188 lesions (57.5%) as other benign lesions, and 22 lesions (6.7%) as malignant.

### 3.4. Benign Lesions

**Figure 2:**
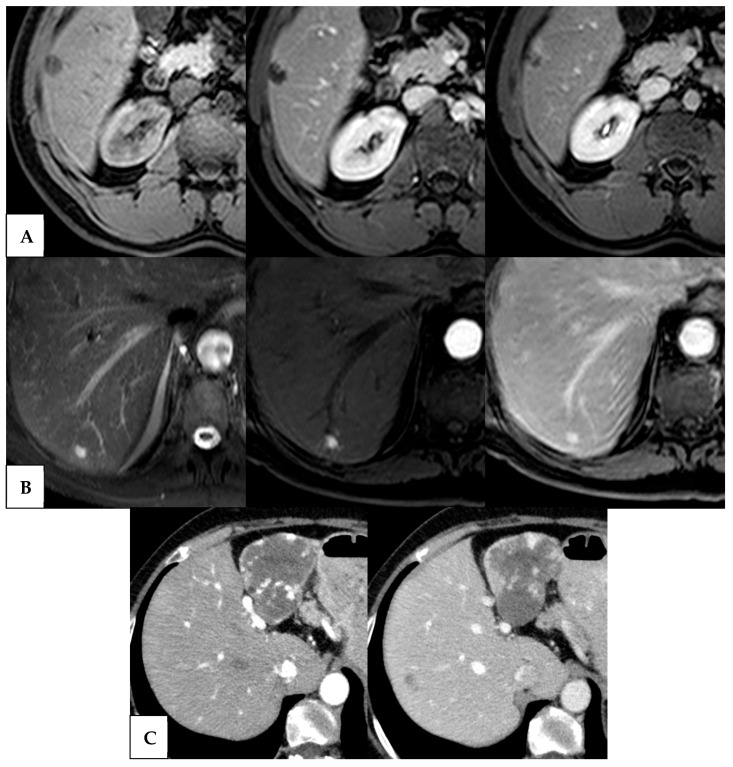
(**A**) MRI scan showing T2-weighted image and contrast-enhanced T1-weighted images (portal and delayed phases) of a slow-filling hemangioma, (**B**) MRI scan showing T2-weighted image and contrast-enhanced T1-weighted images (arterial and portal phases) of a flash-filling hemangioma, and (**C**) CT scan showing arterial and portal phase images of a giant hemangioma.

Of the 305 benign lesions, 117 (38.4%) were confidently diagnosed as hemangiomas. Among these hemangiomas, 38 were giant, 20 slow-filling hemangiomas, 13 flash-filling hemangiomas, and 46 showed no unusual imaging features (Table 3 and Figure 2). All lesions classified as flash-filling hemangiomas met the predefined imaging and clinical criteria, including stability in size on prior or follow-up imaging. Other benign lesions included 9 cases of FNH, 9 cases of focal fat deposition, 3 cysts, and 5 other lesions such as abscesses or adenomyomatosis. In 6 cases, no focal lesion was identified upon further imaging.

The remaining 156 lesions could not be assigned a specific benign diagnosis despite review of the available imaging and clinical data. These lesions were classified as unclassified benign lesions because follow-up imaging demonstrated stability or the clinical record showed no evidence of malignancy during the defined follow-up period. Of these uncharacterized lesions, 36 were initially detected on US, 92 on CT, and 28 on MRI. These lesions were generally small, with an average size of 20.2/21.2 mm (R1/R2). Among those evaluated with contrast-enhanced CT or MRI, 60/65% (R1/R2) exhibited early homogeneous enhancement.

**Figure 3:**
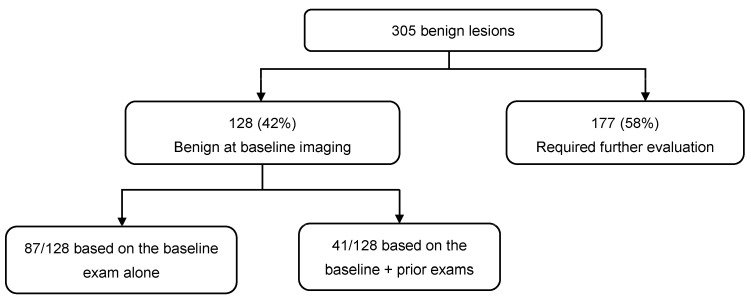
Flowchart illustrating which benign liver lesions could have been retrospectively defined based on baseline imaging alone and which required further evaluation before being confirmed as benign.

On retrospective dedicated review, 128 (42%) of all benign lesions were defined as lesions which could be confidently determined as benign at the index imaging time, either based solely on the index imaging (87/128), or based on a combination of the index and prior imaging studies (41/128), as shown in Figure 3. However, additional diagnostic workup was still recommended in 62 (48.4%) of these 128 benign lesions, including 18 US, 8 CT, 23 MRI, 2 PET-CT, 6 TC-RBC scans, 1 laboratory study, and 8 medical consultations; multiple recommendations were made in 3 cases. Most of the lesions defined benign based solely on the index study (73/87) were hemangiomas, demonstrated predominantly on CT (43 cases), followed by MRI (18 cases) and US (12 cases).

### 3.5. Malignant Lesions

**Figure 4:**
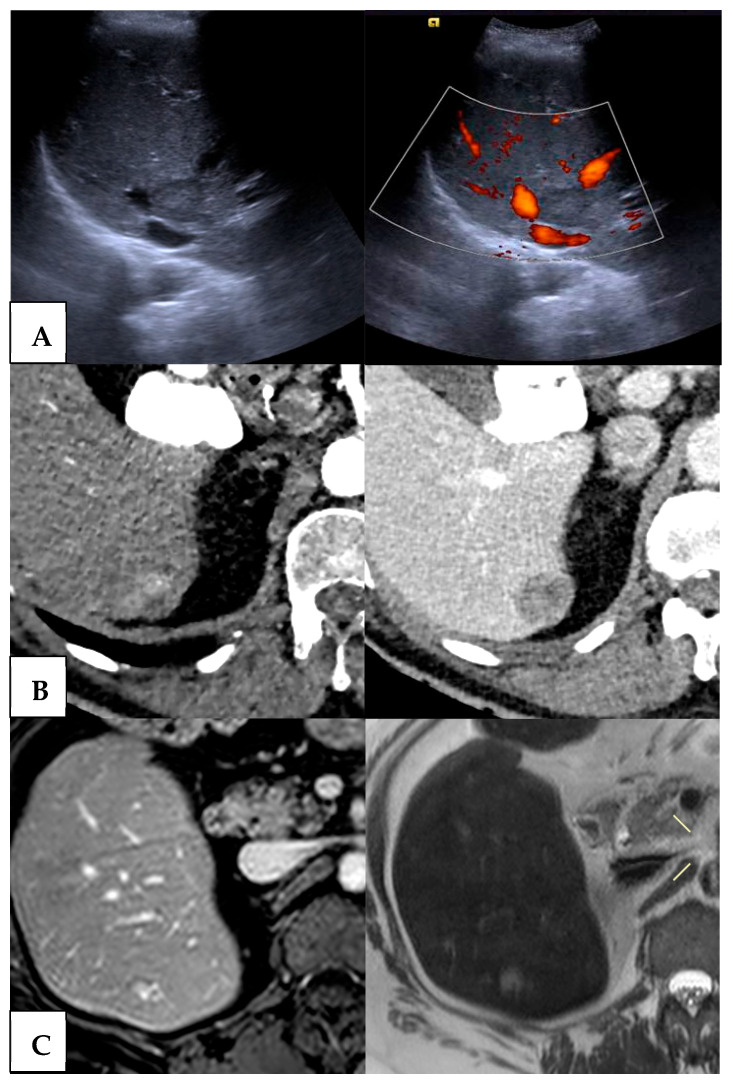
Representative images of liver lesions initially labeled as atypical hemangiomas in radiology reports, later diagnosed as malignant. (**A**) Ultrasound images, including color Doppler, of metastasis of gastrointestinal origin. (**B**) CT scan showing arterial and portal phase images of a neuroendocrine tumor (NET). (**C**) MRI scan showing T1 portal phase and T2-weighted images of liver metastases from breast cancer.

A total of 22 lesions (6.7%) were diagnosed as malignant (Table S2, Supplementary Data, Figure 4). All 22 occurred in different patients; none of the nine patients with repeated qualifying index studies (Section 3.1) had a malignant lesion. These studies were originally interpreted by 15 different radiologists, with 10 studies (45.5%) from the X Medical Center and 12 studies (54.5%) from the Y Medical Center. The frequency of malignant lesions did not differ significantly across imaging modalities (US 8.5%, CT 5.5%, MRI 7.1%; *p* = 0.617).

Malignant lesions included seven cases of hepatocellular carcinoma (HCC) (31.8%), six cases of metastasis from various origins (27.3%), three cases of cholangiocarcinoma (13.6%), two cases of primary hepatic lymphoma (9.1%), two cases of metastatic neuroendocrine tumors (9.1%), one adenocarcinoma likely of ovarian origin (4.5%), and one case of Perivascular Epithelioid Cell tumor (4.5%). Among these cases, 11 (50%) had no recommendations for further workup, and 10 (45%) did not mention malignancy in the differential diagnosis.

### 3.6. Lesion Characteristics

Significant imaging differences between benign and malignant lesions included lesion size, margin definition, enhancement patterns, diffusion restriction, and background liver fibrosis. Malignant lesions were notably larger, with mean sizes of 49.5 mm (R1) and 50.8 mm (R2), compared to benign lesions (28.1 mm, R1; 29.1 mm, R2) (*p* < 0.001 for both readers). Ill-defined borders were significantly more common in malignant lesions (50% R1, *p* = 0.001; 54.5% R2, *p* < 0.001) compared to benign lesions (18.1% R1; 17.9% R2). Early heterogeneous enhancement was significantly more frequent in malignant lesions (46.2% for both readers, *p* < 0.001) versus benign lesions (9.7% R1; 9.3% R2). Among the small subset of lesions evaluated with MRI, restricted diffusion was more frequently observed in malignant than benign lesions (Table 1). However, only four malignant lesions underwent MRI, limiting the strength of this observation. Additionally, moderate-to-severe liver fibrosis was significantly more prevalent in malignant lesions (13.6% for both readers; *p* < 0.001 R1, *p* = 0.002 R2) compared to benign lesions (1.6% R1; 2.0% R2). Detailed comparative imaging features are summarized in Table 1.

There were no statistically significant differences between benign and malignant lesions in terms of hypoechogenicity or absence of the expected progressive centripetal filling of contrast on consecutive phases. Hypoechoic lesions were common in both benign (82.6% R1; 82.2% R2) and malignant lesions (100% R1 and R2) detected on ultrasound, with no significant association (*p* = 0.173 for R1; not applicable for R2). Similarly, the absence of progressive centripetal filling was observed in 19.5% (R1) and 18.8% (R2) of benign lesions compared to 30.8% (R1) and 7.7% (R2) of malignant lesions (*p* = 0.327 and 0.313, respectively). 

### 3.7. Inter-Rater Agreement

Inter-rater agreement was assessed using weighted kappa statistics. Excellent agreement was found for peripheral continuous enhancement (κ = 0.940), early homogeneous enhancement (κ = 0.899), hypoechoic lesion (κ = 0.851), steatotic liver (κ = 0.828), fibrotic liver (κ = 0.819), ill-defined borders (κ = 0.817), and early heterogeneous enhancement (κ = 0.800). Good agreement was observed for additional suspicious lesions (κ = 0.706), centripetal fill-in (κ = 0.789), and restricted diffusion (κ = 0.604). The agreement was moderate for early washout (κ = 0.584). For lesion size, inter-observer agreement was excellent, with an ICC of 0.971 (95% CI: 0.963–0.977, two-way mixed-effects model, absolute agreement, *p* < 0.001).

## 4. Discussion

In this retrospective multicenter study of 327 hepatic lesions initially labeled as “atypical hemangiomas,” the majority (93.3%) were ultimately determined benign; however, a significant proportion (6.7%) proved malignant, likely reflecting a combination of variability in subspecialty expertise and ambiguous terminology. These findings highlight the need for more precise diagnostic definitions and lesion characterization practices. Under a strict lesion-based definition, the malignant lesions were not atypical hemangiomas; rather, they were malignant lesions that had been assigned this reporting label before the final diagnosis was established.

While previous research described atypical hemangiomas with distinctive imaging characteristics, such as echoic borders, rapid filling patterns, calcifications, fluid-fluid levels, and other various morphologies (hyalinized, cystic, pedunculated) [1,5], our study found a broader group of lesions initially labeled atypical, which represents a wide range of benign as well as malignant lesions.

Our findings underscore the clinical implications of misclassification, including both overlooked malignancies and unnecessary follow-up investigations for benign lesions. Malignant lesions in our study were notably larger (mean size: 49.5/50.8 mm) compared to benign lesions (mean size: 28.1/29.1 mm) (R1/R2 respectively) and more frequently exhibited ill-defined margins, early heterogeneous arterial enhancement, and early washout. Restricted diffusion, assessed using DWI together with the corresponding ADC maps, was more frequent among malignant lesions (100% for R1 and 75% for R2). This finding supports the value of diffusion as one component of multiparametric assessment [15]. However, only four malignant lesions underwent MRI in the present cohort, and this observation should therefore be interpreted cautiously and considered hypothesis-generating. Furthermore, diffusion findings should not be interpreted in isolation because T2 shine-through and central sclerosis, scarring, or thrombosis may mimic restriction in hemangiomas. As expected, liver fibrosis and additional suspicious lesions in the liver were two characteristics more commonly found in the malignant lesions group.

Nearly half (62/128, 48.4%) of lesions diagnosable as benign at baseline underwent unnecessary evaluations, including advanced imaging and specialist consultations. Although some required comparison with prior imaging, 87 lesions could be classified as benign on the index study alone, with most (73/87) being classic hemangiomas. This excessive workup elevates healthcare costs and patient anxiety, highlighting an opportunity to improve clinical practice.

A substantial subset of lesions (156/305, 51.1%) classified as benign could not be assigned a specific final diagnosis despite review of the available imaging and clinical data. Their classification was based on longitudinal imaging stability or the absence of clinical evidence of malignancy during follow-up. These lesions should therefore not be interpreted as confirmed atypical hemangiomas or as representing a distinct atypical hemangioma phenotype. Rather, they illustrate the heterogeneous range of indeterminate benign-appearing lesions to which the reporting label “atypical hemangioma” was applied in routine practice.

In light of these findings, we recommend refining diagnostic terminology and standardizing workup strategies. Lesions with classic imaging features should be reported confidently as “hemangioma” without further evaluation. Specific subtypes, such as giant, slow-filling, and flash-filling hemangiomas, should be reported with the subtype specified when supported by the imaging findings and clinical context.

For lesions that cannot be confidently diagnosed, we suggest the use of clear imaging descriptors and specific workup recommendations, including contrast-enhanced US, multiparametric MRI, including hepatobiliary contrast when clinically appropriate, or technetium-99 m-labeled red blood cell scintigraphy. The choice of modality should be tailored to the lesion’s size and imaging appearance, the presence of chronic liver disease or an extrahepatic malignancy, local availability, and radiologist expertise. Importantly, when malignancy cannot be confidently excluded, radiologists should avoid the ambiguous standalone term “atypical hemangioma” and instead use explicit descriptors such as “probably benign,” “suspicious,” or “possibly malignant,” along with a clear management recommendation. Due to the vascular nature of hemangiomas, percutaneous biopsy should generally be avoided while hemangioma remains a plausible diagnosis. If imaging remains indeterminate, dedicated contrast-enhanced imaging, imaging follow-up, or multidisciplinary review should generally precede consideration of tissue sampling. Our study has several limitations, including its retrospective design and variable diagnostic pathways across patients. A key limitation is that 156 lesions classified as benign could not be assigned a specific final diagnosis. Benignity was inferred from imaging stability or the absence of clinical evidence of malignancy during follow-up rather than from definitive imaging characterization or histopathologic confirmation. Although the minimum follow-up period was two years, the median and mean follow-up durations exceeded five years, providing reasonable reassurance. This limits our ability to determine the exact spectrum of benign entities included in this subgroup or to establish whether they represented true atypical hemangiomas, other benign focal lesions, or nonspecific imaging abnormalities. However, this uncertainty also reflects the real-world use of the reporting label that this study was designed to evaluate. Additionally, the multicenter design, while enhancing generalizability, inherently introduces variability among radiologists’ diagnostic thresholds. Finally, we did not evaluate the subspecialty training of the interpreting radiologists, which could influence diagnostic accuracy. Radiologists specializing in abdominal imaging might more consistently recognize hemangioma variants than non-subspecialized readers. Nevertheless, this limitation reflects real-world practice, as abdominal imaging studies are frequently interpreted by radiologists without specific expertise in liver imaging, leading to interpretive inconsistencies, including major discrepancy rates of 26–32% in abdominopelvic CT interpretation and increased discordance when studies are interpreted outside one’s subspecialty [8,9,10,11,12]. These findings further underscore the importance of accurate, standardized terminology to support clear communication and optimal patient management. We did not perform modality-stratified analyses because of the limited number of malignant lesions within each modality, particularly MRI, which would have produced unstable subgroup estimates. Larger studies are needed to evaluate modality-specific differences separately. Future prospective studies should incorporate radiologists’ subspecialties, standardized imaging criteria, and advanced analytical tools such as artificial intelligence or predictive modeling to further enhance diagnostic accuracy.

## 5. Conclusions

The term “atypical hemangioma” should be reserved for lesions ultimately confirmed as hemangiomas that lack classic imaging characteristics. In our cohort, broader use of this reporting label was associated with suboptimal patient management, including both over-investigation of benign lesions and missed malignancies. Precise terminology and risk-stratified workup protocols may improve patient management and reduce diagnostic uncertainty.

## Figures and Tables

**Table 1:** Comparative analysis of key imaging characteristics between benign and malignant hepatic lesions, including size, enhancement patterns, and diffusion restriction.

Lesion Characteristics					
SD		Benign	Malignant	Overall	*p* Value
Size R1 mm	Mean (median, SD)	28.1 (20, ±23)	49.5 (40, ±38)	29.6 (20, ±26)	<0.001
Size R2 mm	Mean (median, SD)	29.1 (20.5, ±25)	50.8 (39.5, ±37)	30.6 (20, ± 27)	<0.001
Ill-defined borders R1	yes	54 (18.1%)	11 (50.0%)	65 (20.2%)	0.001
	no	242 (80.9%)	11 (50.0%)	253 (78.8%)	
	N/A	3 (1.0%)	0 (0.0%)	3 (0.9%)	
Ill-defined borders R2	yes	53 (17.9%)	12 (54.5%)	65 (20.4%)	<0.001
	no	240 (81.1%)	10 (45.5%)	250 (78.6%)	
	N/A	3 (1.0%)	0 (0.0%)	3 (0.9%)	
Peripheral continuous enhancement R1	yes	12 (5.8%)	5 (38.5%)	17 (7.8%)	0.037
	no	192 (93.2%)	8 (61.5%)	200 (91.3%)	
	N/A	2 (1.0%)	0 (0.0%)	2 (0.9%)	
Peripheral continuous enhancement R2	yes	13 (6.3%)	5 (38.5%)	18 (8.3%)	<0.001
	no	192 (93.7%)	8 (61.5%)	200 (91.7%)	
	N/A	0 (0%)	0 (0%)	0 (0%)	
Early homogeneous enhancement R1	yes	97 (46.9%)	1 (7.7%)	98 (44.5%)	0.021
	no	109 (52.7%)	12 (92.3%)	121 (55.0%)	
	N/A	1 (0.5%)	0 (0.0%)	1 (0.5%)	
Early homogeneous enhancement R2	yes	94 (45.6%)	1 (7.7%)	95 (43.4%)	0.007
	no	112 (54.4%)	12 (92.3%)	124 (56.6%)	
	N/A	0 (0%)	0 (0%)	0 (0%)	
Early heterogeneous enhancement R1	yes	20 (9.7%)	6 (46.2%)	26 (11.8%)	<0.001
	no	186 (89.9%)	7 (53.8%)	193 (87.7%)	
	N/A	1 (0.5%)	0 (0.0%)	1 (0.5%)	
Early heterogeneous enhancement R2	yes	19 (9.3%)	6 (46.2%)	25 (11.5%)	<0.001
	no	185 (90.7%)	7 (53.8%)	192 (88.5%)	
	N/A	0 (0%)	0 (0%)	0 (0%)	
Early washout R1	yes	0 (0.0%)	1 (7.7%)	1 (0.5%)	<0.001
	no	187 (90.3%)	11 (84.6%)	198 (90.0%)	
	N/A	20 (9.7%)	1 (7.7%)	21 (9.5%)	
Early washout R2	yes	0 (0.0%)	2 (15.4%)	2 (0.9%)	<0.001
	no	194 (95.6%)	11 (84.6%)	205 (94.9%)	
	N/A	9 (4.4%)	0 (0.0%)	9 (4.2%)	
Restricted diffusion R1	yes	9 (17.6%)	4 (100.0%)	13 (23.6%)	<0.001
	no	41 (80.4%)	0 (0.0%)	41 (74.5%)	
	N/A	1 (2.0%)	0 (0.0%)	1 (1.8%)	
Restricted diffusion R2	yes	4 (7.8%)	3 (75.0%)	7 (12.7%)	<0.001
	no	47 (92.2%)	1 (25.0%)	48 (87.3%)	
	N/A	0 (0%)	0 (0%)	0 (0%)	

R1, reader 1; R2, reader 2. Enhancement characteristics were assessed only on examinations in which enhancement could be evaluated (contrast-enhanced CT, MRI, or contrast-enhanced ultrasound, when available). Restricted diffusion was assessed only on MRI. Percentages are calculated using the number of evaluable examinations for each imaging feature; therefore, denominators vary according to imaging modality, feature availability, and reader-specific assessability.

**Table 2:** Summary of patient demographics, index imaging modalities, and institutional distribution of the 327 included cases.

Patient Demographics Overall and Divided by Lesion Nature					
		Benign	Malignant	Overall (Benign + Malignant Lesions)	*p* Value
Patient Characteristics					
Age	Mean (median, SD)	57.3 (58, ±16)	63.9 (63, ±16)	57.8 (59, ±16)	0.067
Female		153 (50.2%)	10 (45.5%)	163 (49.8%)	0.670
Modality	US	97 (31.8%)	9 (40.9%)	106 (32.4%)	0.617
	CT	156 (51.1%)	9 (40.9%)	165 (50.5%)	
	MRI	52 (17.0%)	4 (18.2%)	56 (17.1%)	

**Table 3:** Imaging presentations of confirmed hemangiomas with non-classical or subtype-specific features. Numbers represent mutually exclusive final classifications. An additional 46 confirmed hemangiomas showed no unusual imaging features and are not included in the table.

Imaging Presentation	Number	Defining Characteristics
Giant hemangioma	38	Diameter greater than 4 cm; may show heterogeneous appearance related to internal degeneration
Slow-filling hemangioma	20	Delayed progressive enhancement with slow centripetal filling
Flash-filling hemangioma	13	Small lesion with homogeneous arterial enhancement, no washout, persistent blood-pool-like enhancement, appropriate clinical context, and imaging stability

## Data Availability

The data presented in this study are not publicly available due to privacy and ethical restrictions. Data may be available from the corresponding author upon reasonable request and subject to approval by the participating institutions.

## Supplementary Materials

The following supporting information can be downloaded at https://www.mdpi.com/article/10.3390/jcm15155923/s1: Table S1: Background Liver Characteristics of Benign and Malignant Hepatic Lesions Initially Labeled as “Atypical Hemangiomas”; Table S2: Demographic, Imaging, and Diagnostic Characteristics of Patients with Malignant Hepatic Lesions Initially Labeled as “Atypical Hemangiomas”.

## Abbreviations

R1, reader 1; R2 reader 2; DWI, diffusion-weighted imaging; ADC, apparent diffusion coefficient; FNH, focal nodular hyperplasia; HCC, hepatocellular carcinoma; PECOMA, perivascular epithelioid cell tumor; LI-RADS, Liver Imaging Reporting and Data System.

## References

[1] Vilgrain V., Boulos L., Vullierme M.-P., Denys A., Terris B., Menu Y. (2000). Imaging of Atypical Hemangiomas of the Liver with Pathologic Correlation. RadioGraphics.

[2] Bajenaru N., Balaban V., Săvulescu F., Campeanu I., Patrascu T. (2015). Hepatic hemangioma-review. J. Med. Life.

[3] Jang H.-J., Kim T.K., Lim H.K., Park S.J., Sim J.S., Kim H.Y., Lee J.-H. (2003). Hepatic Hemangioma: Atypical Appearances on CT, MR Imaging, and Sonography. Am. J. Roentgenol..

[4] Blumhagen N., Fisher K.L. (2017). Atypical Hepatic Hemangioma: A Case Study. J. Diagn. Med. Sonogr..

[5] Vásquez Montoya J.D., Molinares B., Vásquez Trespalacios E.M., García V., Pérez Cadavid J.C. (2018). Atypical hepatic haemangiomas. BJR|Case Rep..

[6] Lin S., Zhang L., Li M., Cheng Q., Zhang L., Zheng S. (2017). Atypical hemangioma mimicking mixed hepatocellular cholangiocarcinoma: Case report. Medicine.

[7] Yıldırım M.B., Şahiner İ.T., Poyanlı A., Acunaş B., Güllüoǧlu M., İbiş C., Tekant Y., Özden I. (2021). Malignant Tumors Misdiagnosed as Liver Hemangiomas. Front. Surg..

[8] Yesilagac H., Arer I.M., Gulalp B., Yabanoglu H., Karagun O., Karadeli E. (2020). Generalist versus Abdominal Subspecialist Radiologist Interpretations of Abdominopelvic Computed Tomography Performed on Patients with Abdominal Pain and its Impact on the Therapeutic Approach. Adv. J. Emerg. Med..

[9] Rich N.E., Chernyak V. (2023). Standardizing liver imaging reporting and interpretation: LI-RADS and beyond. Hepatol. Commun..

[10] Corwin M.T., Lee A.Y., Fananapazir G., Loehfelm T.W., Sarkar S., Sirlin C.B. (2018). Nonstandardized Terminology to Describe Focal Liver Lesions in Patients at Risk for Hepatocellular Carcinoma: Implications Regarding Clinical Communication. Am. J. Roentgenol..

[11] Cox V., Haddad A., Lendoire M., Yedururi S., Marcal L., Panettieri E., Kawaguchi Y., Bassett R., Vauthey J.-N., Kang H.C. (2025). Impact of Imaging Review and Multidisciplinary Discussion During Hepatobiliary Tumor Board: A Prospective Study. Ann. Surg. Oncol..

[12] Chung R., Rosenkrantz A.B., Shanbhogue K.P. (2020). Expert radiologist review at a hepatobiliary multidisciplinary tumor board: Impact on patient management. Abdom. Radiol..

[13] Harris P.A., Taylor R., Thielke R., Payne J., Gonzalez N., Conde J.G. (2009). Research electronic data capture (REDCap)—A metadata-driven methodology and workflow process for providing translational research informatics support. J. BioMed Inf..

[14] Harris P.A., Taylor R., Minor B.L., Elliott V., Fernandez M., O’Neal L., McLeod L., Delacqua G., Delacqua F., Kirby J. (2019). The REDCap consortium: Building an international community of software platform partners. J. BioMed Inf..

[15] Wang H., Guo S. (2023). Solitary hypovascular hepatic nodules: Comparison and differentiation between peripheral nodular cholangiocarcinoma and atypical liver hemangioma with MRI. Transl. Cancer Res..

