# Supplementary material for: Use of the Term “Atypical Hepatic Hemangioma” in Radiology Reports: An Analysis of Diagnostic Precision
Source: J Clin Med. 2026 Jul 29;15(15):5923. doi: 10.3390/jcm15155923 (PMC13466356; doi:10.3390/jcm15155923)
Supplement: Supplementary file 1 [file jcm-15-05923-s001.zip › jcm-4406560-supplementary.pdf]

Supplementary Data

Table S1: Background Liver Characteristics of Benign and Malignant Hepatic Lesions Initially Labeled as "Atypical Hemangiomas"

Background Liver Characteristics					
		Benign (% within benign lesions)	Malignant (% within malignant lesions)	Overall (% of overall lesions)	p value
Additional suspicious lesions R1	yes	22 (7.5%)	5 (22.7%)	27 (8.6%)	0.045
	no	268 (91.5%)	17 (77.3%)	285 (90.5%)	
	N/A	3 (1.0%)	0 (0.0%)	3 (1.0%)	
Additional suspicious lesions R2	yes	24 (8.2%)	6 (27.3%)	30 (9.5%)	0.012
	no	266 (90.5%)	16 (72.7%)	282 (89.2%)	
	N/A	4 (1.4%)	0 (0.0%)	4 (1.3%)	
Fatty liver R1	None/Mild	234 (77.0%)	20 (90.9%)	254 (77.9%)	0.128
	Moderate-severe	70 (23.0%)	2 (9.1%)	72 (22.1%)	
Fatty liver R2	None/Mild	237 (78.0%)	20 (90.9%)	257 (78.8%)	0.151
	Moderate-severe	67 (22.0%)	2 (9.1%)	69 (21.2%)	
Fibrotic liver R1	None/mild	300 (98.4%)	19 (86.4%)	319 (97.6%)	<0.001
	Moderate-severe	5 (1.6%)	3 (13.6%)	8 (2.4%)	
Fibrotic liver R2	None/mild	289 (98.0%)	19 (86.4%)	308 (97.2%)	0.002
	Moderate-severe	6 (2.0%)	3 (13.6%)	9 (2.8%)	

Table S2. Demographic, Imaging, and Diagnostic Characteristics of Patients with Malignant Hepatic Lesions Initially Labeled as "Atypical Hemangiomas".

Demographic and pathological data of patients with malignant lesions					
	Diagnosis	Ground truth established by	Gender	Age	Modality
1	PECOMA	Tissue diagnosis	Male	68	US
2	HCC	Tissue diagnosis	Male	53	US
3	Metastasis gastrointestinal origin	Tissue diagnosis	Male	42	US
4	HCC	Imaging (LIRADS 5 in cirrhotic liver)	Male	87	US
5	HCC	Tissue diagnosis	Male	67	US
6	Metastatic Neuroendocrine tumor	Tissue diagnosis	Male	63	US
7	HCC	Imaging (LIRADS 5 in cirrhotic liver)	Male	91	US
8	HCC Vs. Cholangiocarcinoma	Imaging. Patient passed soon after imaging	Male	82	CT
9	PECOMA	Tissue diagnosis	Male	61	CT
10	Metastasis gastrointestinal origin	Tissue diagnosis	Male	62	CT
11	Cholangiocarcinoma	Tissue diagnosis	Male	67	CT
12	Liver lymphoma	Tissue diagnosis	Male	63	MRI
13	Metastasis breast imaging, follow up	Tissue diagnosis	Female	58	US
14	Liver lymphoma	Tissue diagnosis	Female	29	US
15	Metastatic Neuroendocrine tumor	Tissue diagnosis	Female	75	CT
16	Most probably ovarian origin	New lesion in metastatic ovarian cancer	Female	85	CT
17	Metastatic Pancreaticobilliary origin	Tissue diagnosis	Female	50	CT
18	Metastasis gastrointestinal origin	Tissue diagnosis	Female	86	CT
19	Most probably cholangiocarcinoma	Imaging. Patient passed soon after imaging	Female	40	CT
20	Metastasis breast imaging	Tissue diagnosis	Female	55	MRI
21	Metastasis gastrointestinal origin	Tissue diagnosis	Female	57	MRI
22	Transplanted liver, HCC	Tissue diagnosis	Female	66	MRI
